# Unintentional Interpersonal Synchronization Represented as a Reciprocal Visuo-Postural Feedback System: A Multivariate Autoregressive Modeling Approach

**DOI:** 10.1371/journal.pone.0137126

**Published:** 2015-09-23

**Authors:** Shuntaro Okazaki, Masako Hirotani, Takahiko Koike, Jorge Bosch-Bayard, Haruka K. Takahashi, Maho Hashiguchi, Norihiro Sadato

**Affiliations:** 1 Division of Cerebral Integration, National Institute for Physiological Sciences, Okazaki, Aichi, Japan; 2 School of Linguistics and Language Studies, and Institute of Cognitive Science, Carleton University, Ottawa, Ontario, Canada; 3 Unity of Neurodevelopment, Institute of Neurobiology, UNAM, Campus Juriquilla, Santiago de Querétaro, Querétaro, México; 4 Department of Physiological Sciences, SOKENDAI (The Graduate University for Advanced Studies), Hayama, Kanagawa, Japan; IIT—Italian Institute of Technology, ITALY

## Abstract

People’s behaviors synchronize. It is difficult, however, to determine whether synchronized behaviors occur in a mutual direction—two individuals influencing one another—or in one direction—one individual leading the other, and what the underlying mechanism for synchronization is. To answer these questions, we hypothesized a non-leader-follower postural sway synchronization, caused by a reciprocal visuo-postural feedback system operating on pairs of individuals, and tested that hypothesis both experimentally and via simulation. In the behavioral experiment, 22 participant pairs stood face to face either 20 or 70 cm away from each other wearing glasses with or without vision blocking lenses. The existence and direction of visual information exchanged between pairs of participants were systematically manipulated. The time series data for the postural sway of these pairs were recorded and analyzed with cross correlation and causality. Results of cross correlation showed that postural sway of paired participants was synchronized, with a shorter time lag when participant pairs could see one another’s head motion than when one of the participants was blindfolded. In addition, there was less of a time lag in the observed synchronization when the distance between participant pairs was smaller. As for the causality analysis, noise contribution ratio (NCR), the measure of influence using a multivariate autoregressive model, was also computed to identify the degree to which one’s postural sway is explained by that of the other’s and how visual information (sighted vs. blindfolded) interacts with paired participants’ postural sway. It was found that for synchronization to take place, it is crucial that paired participants be sighted and exert equal influence on one another by simultaneously exchanging visual information. Furthermore, a simulation for the proposed system with a wider range of visual input showed a pattern of results similar to the behavioral results.

## Introduction

Synchronization is defined as spontaneous pattern formation, and has been accounted for by the “self-organization” system, a mechanism that uses coupled oscillators and is characterized by its nonlinear dynamics [1,2]. Such pattern formation has been observed in many different social, physical, and biological systems [3,4]. In human behaviors, at least, the precise underlying mechanism for synchronization is still unclear. As mentioned in Oullier et al. (2008) [5], it is unclear whether coupled oscillators are required to produce synchronized behaviors and events (e.g., two individuals equally contributing to synchronization, one individual serving as a driving force for synchronization). Importantly, in some social events, synchronization takes place not only without the intention of the individuals involved, but also without a time lag; thus, an account that explains spontaneity in synchronization is needed. In addition, there is a methodological challenge that accompanies testing hypothesized systems for synchronization (for both experimental set-ups and analysis methods); Synchronized behaviors and events are often fluid, i.e., constantly changing, and can be influenced by unknown factors. The goal of the present paper is to provide a new account for synchronized phenomena. Specifically, this paper proposes a visuo-postural feedback system that operates on two individuals, in which the timing of synchronization depends on the degree of influence of visual input exchanged between the individuals. By carrying out a well controlled experiment, data analyses with cross correlation and causality, and a simulation, we demonstrate that such a feedback system is sufficient to explain the synchronization of postural sway between paired individuals.

Exploration of the mechanism underlying social synchronization is important in understanding human social behavior. In social activities, such as dancing with a partner, moving a heavy table together, and playing team sports, interpersonal behavioral synchronization is achieved without verbal communication and often occurs in both form, i.e., the behaviors or actions that are matched, and their timing [3]. It seems that humans are equipped with a tendency, perhaps, from birth, to imitate or mimic other individuals’ manners or postural movements without intention (or unconsciously). It has been reported that individuals unconsciously and automatically imitate other people’s behaviors such as facial expressions, foot shakes, and hand positions [6–10]. These phenomena of “mimicry” may have developed as part of human evolution. Hattori et al. 2013 [11] describe a chimpanzee that, after some training, managed to align its finger tapping rhythm to auditory stimuli. Other species such as birds show vocal synchronization with others of their kind [12]. Thus, synchronization can be, in part, viewed as mimicry, which may serve as a basis for a wide range of synchronized phenomena in human behavior. Mimicry is focused on synchronized forms or manners of behaviors or actions, and thus, the timing of synchronization is often viewed as irrelevant. Some studies on this topic even suggest that shared behaviors are triggered more often when there is a longer time lag after an individual’s initial behavior occurs before his or her partner’s behavior takes place [13]. It seems that mimicry or the “chameleon effect” as it is sometimes called, is a natural part of people’s behavior and likely occurs when they do not notice anything special or extraordinary [6,9]. Chartrand & Bargh (1999) [6] proposed that a linkage between perception and behavior plays a key role in mimicry. Their insight into the mimicry mechanism has relevance to motor-based theories of synchronization for human behavior.

More recent studies highlight the need to investigate the timing of synchronized behaviors and actions in order to better understand human social behavior. As mentioned already, in the studies on mimicry, the timing on imitated behaviors is not likely to be critical. In contrast, rhythmic actions or motions shared by two individuals looking at each other are often entrained into in-phase unintentional synchronization (e.g., [5,14–16]). Recent studies have shown that matched timing as well as matched forms of movement induce higher ratings of rapport [17] and liking [18] towards one’s partner, and increase cognitive capacities such as remembering the face and utterances of a partner with whom one interacted [19]. Furthermore, it has been reported that synchronization of more than one action or type of movement occurs simultaneously between individuals partaking dynamic, higher-order cognitive and social activities. For example, Shockley et al., 2003 [20] and 2009 [21] found that during cooperative conversation, the eye gaze and postural sway of the individuals taking part in conversation can synchronize, in addition to linguistic factors such as the choice of words and speech rate. Their study implies that one social factor influences another social factor and underscores the importance of looking into the entire social event as a whole.

Previous researchers have argued that an essential component of real-time social interactions is reciprocal coupling via perceptual-motor linkages between interacting individuals [22–26]. In addition, recent well controlled studies have suggested that reciprocal coupling of the reaction to and the prediction of a partner’s action is the basis of joint actions with either a short time lag or no time lag at all. Those studies demonstrate that conscious, intentional and active involvement of individuals in synchronized events is crucial. Konvalinka et al. (2010) [16] showed that the interpersonal synchronization of rhythmic tapping motions took place with less variant time lag when the interacting individuals could hear their partner’s tapping sounds than when they could not. When they could, both members of these tapping pairs showed the “follower pattern”, meaning that they followed their partner’s actions and vice versa. In order to follow a partner’s actions, it is essential that individuals predict their actions. Thus, based on Konvalinka et al.’s findings, we can conclude that interpersonal in-phase synchronization can be facilitated by the mutual ability to predict the other’s subsequent action. Similar to Konvalinka et al. [16], Noy et al. (2011) [27] have also shown the importance of predicting their partner’s actions in relation to the timing of synchronization. Specifically, they investigated improvised movement using a synchronized lever swinging task, in which synchronization was defined as obtaining a small, mean relative difference in velocity and timing between zero-velocity events. They replicated this phenomenon using a computational model with reciprocally coupled controllers that react to and predict another’s action.

Considering the spontaneity of social entrainment [5], social synchronization may occur without any of the predictors suggested by the studies mentioned above [16,27]. To test this possibility, we developed a behavioral experiment that to the best of our knowledge, satisfies the factors that were missing in all of the previous studies. We collected time series data for postural sway of two individuals standing face to face. We controlled the participants’ visual input. One’s vision is known to control his or her standing posture by generating postural reactions that help to stabilize the individuals with respect to the visual world (see [28] for anterior-posterior (AP) axis; see [29] for left-right (LR) axis). Compared to the situations in which individuals’ eyes are closed, the amount of postural sway is reduced by half when their eyes were open [30,31]. This indicates the prominent influence of visual input on one’s postural control. Since the natural and unintentional postural sway of an individual is fed back to the partner through his or her vision, the experimental setting of the present study (see below) is ideal to evaluate the reciprocal inter-personal interaction with minimum task effort.

Our hypothesis was that a reciprocal and equivalent influence between visuo-motor linkages between two individuals induces “lag-0 synchronization” of unintentional, non-rhythmic behavior (i.e., postural sway). Lag-0 synchronization is achieved when there is no time lag in matched behaviors between pairs of individuals. To test our hypothesis, cross correlation was applied as an analysis method to look into the relation between the timing of the synchronization of postural sway and the exchange of the type of visual input between paired individuals. To further test our hypothesis we dissociated the influence between paired participants using autoregressive (MVAR) model estimation and a causality analysis [32–34]. Finally, the time series of postural sway of the two individuals were simulated based on the estimated MVAR model. The results of the present study support our hypothesis, suggesting the significance of timing to individuals engaged in reciprocal interaction for lag-0 synchronization of postural sway.

## Materials and Methods

The aim of the present paper is to investigate whether or not postural sway is subject to lag-0 synchronization between two individuals and if so, to determine its underlying mechanisms. To this end, 1) a behavioral experiment, and 2) a model simulation with the use of a multivariate autoregressive model, were conducted.

### Participants

A total of 44 females (mean age ± standard deviation = 25.0 ± 7.4 years old) participated in a behavioral experiment that investigated postural sway between two individuals. All participants had normal hearing and normal or corrected-to-normal vision. None of the participants had a history of neurological, psychiatric, or sleep disorders. Each participant was randomly paired with another participant, resulting in a total of 22 pairs. Prior to the experiment, we ensured that pairs were not mutually acquainted. The study was approved by the ethical committee of the National Institute for Physiological Sciences, Japan and conducted according to the Declaration of Helsinki. All participants gave written informed consent before the experiment started.

### Procedures

**Fig 1:**
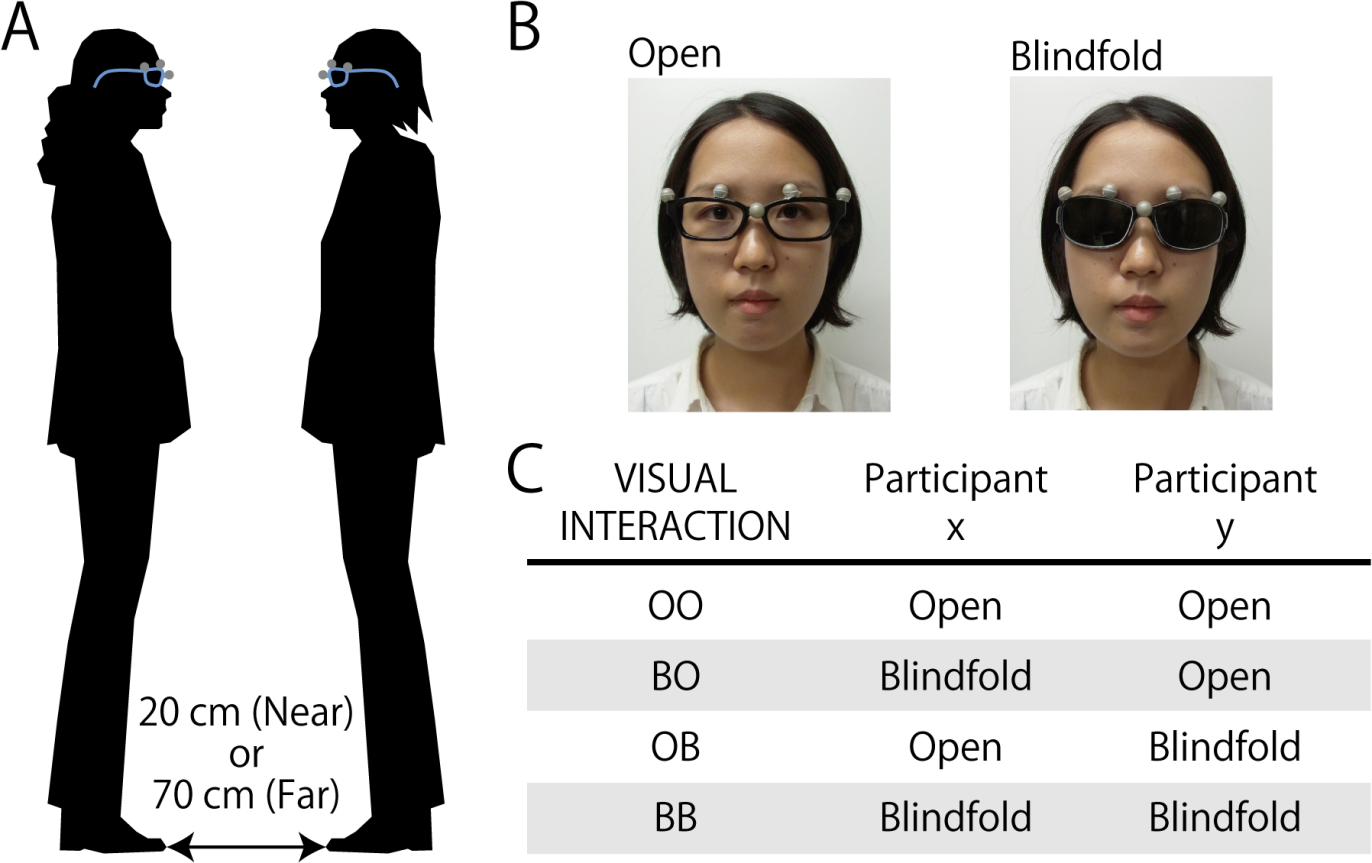
Test conditions for behavioral experiment. (A) In the behavioral experiment, pairs of female participants were instructed to stand upright, in silence, facing each other at a toe-to-toe distance of 20 cm (Near) or 70 cm (Far), with their vision enabled (Open) or blocked (Blindfold). (B) Participants wore examiner-provided glasses with no lenses for the Open condition (left panel) or opaque lenses for the Blindfold condition (right panel). Glasses were equipped with a motion capture system (5 gray markers) so that on-going changes in participants’ head position (i.e., postural sway) were recorded while the task illustrated in (A) was performed. (C) Paired participants (x, y) carried out the task illustrated in (A) with four VISUAL INTERACTION conditions for each DISTANCE condition (Near, Far) (not shown here; see (A)): Open-Open (OO), Blindfold-Open (BO), Open-Blindfold (OB), and Blindfold-Blindfold (BB).

Pairs of participants were instructed to wear glasses and stand upright face to face in the middle of a square, sound attenuated room (310 cm × 310 cm). Using a 2 × 4 design with the first factor DISTANCE (Near, Far) and the second factor VISUAL INTERACTION ((Eyes-)Open-Open (OO), Blindfold-Open (BO), Open-Blindfold (OB), Blindfold-Blindfold (BB)) (see Fig 1), each pair of participants stood either 20 cm (Near) or 70 cm (Far) away from each other (Note: The individual that appears in Fig 1 in this paper has given written informed consent, as outlined in PLOS consent form, to publish the figure). The glasses worn by the participants had either a frame without lenses to enable their vision (Open condition) or opaque lenses attached to the frame to block their vision (Blindfold condition). If participants wore glasses in their daily lives and brought them with them, they wore their own glasses for the Open condition. For the Blindfold condition, these participants wore the experimenter-provided glasses with opaque lenses. Participants were asked to remain silent and wear ear plugs to reduce any ambient noise. Each trial lasted 60 seconds, during which participants stood facing their partners in a manner dictated by one of the four conditions of VISUAL INTERACTION. Eight counter-balancing lists constructed based on the VISUAL INTERACTION conditions were pseudo-randomized in blocks based on the DISTANCE conditions and randomly assigned to each pair of participants. Each paired participants completed a total of 32 trials, consisting of 16 trials of the Near DISTANCE condition and another 16 trials of the Far DISTANCE condition. During the experimental session, participants were instructed to look at their partner’s eyes for the Open condition or to look in the direction of their partner’s eyes for the Blindfold condition. Participants were asked not to close their eyes, except for blinks, for both Open and Blindfold conditions. Also, they were told to move or not to move their body and to hold still the position of their head as much as possible. In addition, while standing face to face, they were asked to think about their partner, not something else. They were explicitly asked not to count in their heads or think about the duration of the elapsed time. More than a half of the participants (i.e., 12 of 22 pairs) filled out the optional questionnaire asking whether they were aware of their own postural sway or that of their partner. They were also asked if they recognized whether their postural sway was either synchronized or asynchronized with that of their partner, and whether they intentionally attempted to either synchronize or asynchronize their postural sway with that of their partner. An experimental session lasted approximately 90 minutes, including instructions, optional questionnaire, and debriefing. A short break was provided if needed.

### Recording Device

The experimental room was equipped with eight infrared cameras with a light source (MCU240, Qualisys AB, Sweden). Cameras were suspended from the ceiling of the room at an approximately equal distance from one another, enabling the upper body of the participants to be captured. Five markers attached to the participants’ glasses (at the center of the frame and both upper left and right corners of where each of the lenses would appear in regular glasses) recorded their head position and movement. The markers were used as rigid identifiers for the head position of the participants. Using analysis software (Qualisys Track Manager, Qualisys AB, Sweden), participants’ head position and movement were calculated. We treated the recorded position of the participants’ head as the recorded position of their body. The three spatial axes of the motion capturing fields were calibrated before each experimental session. Following previous studies (e.g., [35,36]), only the data for the spatial position along the anterior-posterior (AP) and left-right (LR) axes, but not the rostro-caudal (RC), i.e., vertical, axis, were analyzed, as postural sway does not usually occur along the RC axis. (Note: For the reader interested in the signal amplitude of postural sway, see S1 Table.) All data were recorded with a sampling rate of 200 Hz.

### Data Analysis

Motion captured time series data for participants’ postural sway were analyzed using three different analysis methods: 1) a cross correlation analysis, and 2) a causality analysis with a multivariate autoregressive (MVAR) model, and 3) a single regression analysis. The cross correlation analysis allowed us to examine the relation between paired participant’s postural sway and the time lag (ms) in the synchronization of postural sway in each of the VISUAL INTERACTION conditions. The causality analysis enabled investigation of the degree to which participants exerted influence on their partner’s postural sway. The analysis was based on the estimation carried out by computing how much of the noise variance (i.e., the noise that was estimated as residual or “driving” noise for the MVAR model) lies in each participant’s postural sway and how much the noise variance of each participant contributes to the postural sway of the paired participants. Finally, a single regression analysis was performed for any time lag calculated in the cross correlation analysis and the degree of bidirectional influence calculated in the causality analysis. This comprehensive analysis examined the relation between the degree of influence between paired sighted participants and the time lag in synchronization of the postural sway.

#### Cross correlation

For the present study, the Eq in (1) was used. In Eq (1), $\bar{x}$ and $\bar{y}$, and *σ*_*x*_ and *σ*_*y*_ indicate the mean and standard deviation of the time series signals, x(*t*) and y(*t*), respectively. *N*_*t*_ (Number of time points) was 12000 (60 seconds × 200 Hz). Using the Eq in (1), for each VISUAL INTERACTION condition (see above), cross correlation was calculated for each trial for each data set of the two time series signals, x(*t*) and y(*t*) before the correlation values were averaged over all trials for all the data sets.

$$R_{\mathrm{xy}}(\tau )=\frac{1}{N_{t}-1}\sum_{t}^{N_{t}-\tau }\frac{\left(x(t+\tau )-\bar{x})(y(t)-\bar{y}\right)}{\sigma_{x}\sigma_{y}}$$ (1)

The linear trend of time series signals (x(*t*) and y(*t*)) was excluded using the “detrend” function of MATLAB (The MathWorks Inc, USA) and the signals were filtered using Hanning windows of the same size as the data length before their cross correlation was calculated (the MATLAB’s “detrend” function computes the least-squares fit of a straight line to the data in question and subtracts the resulting function from the data). A correlation value for the two signals was obtained for each shift of a time point of τ. For the three VISUAL INTERACTION conditions (OO, BO, OB), in the Near DISTANCE condition, the maximum values of correlation were mainly found around 0 second time lag, i.e., from-1 to 1 second. Therefore, the data analyses conducted in this paper were focused on the time points ranging from-1 to 1 second. Here “0 second” refers to “lag-0” or full synchronization between the paired participants, i.e., no delay in the timing of postural sway between the paired participants. Likewise, “-200 (or 200) milliseconds” is interpreted to mean that the postural sway of one of the participants preceded (or was followed by) that of her partner by 200 milliseconds. Data for postural sway were analyzed separately for AP and LR axes. Analyses were carried out using “xcov (cross-covariance)” included in the Signal Processing Toolbox of MATLAB. The time lag (ms) with respect to lag-0 synchronization detected by the cross correlation curves was tested against 0 ms (i.e., lag-0) for the OO condition with one-sample *t*-tests. The time lags in the BO and OB conditions were tested against those in the OO condition with paired *t*-tests using Bonferroni correction (number of comparisons = 2). Finally, only effects that approached significance (*p* < 0.05) are reported in the Results section.

#### Multivariate autoregressive model estimation and causality analysis

Using an MVAR model, we computed the noise contribution ratio (NCR), an index representing the degree of influence between two participants [32–34]. From the analysis options available to us that result in the same computational output (i.e., the Granger causality test with the recent developments, e.g., [37]), we chose Akaike causality (see Ozaki, 2012 [33] for the comparison between the two analysis methods). We did so because this analysis method takes the power spectrums into account and is focused on computing the degree of influence between the variables we are interested in. We use the term “causality” with the following strict definition in this paper: Causality refers to the degree to which one time series data can predict another time series data. This analysis is useful because it allows us to infer the relation between two time series data. An MVAR model, specifically, a bi-variate AR model such as the one used in this study, is a mathematical model of two time series data that can be estimated using the linear sum of the history of the two time series data with the Eqs in (2) and (3). *a*_*i*_, *b*_*i*_, *c*_*i*_, and *d*_*i*_ indicate AR coefficients and *u*_*x*_(*t*) and *u*_*y*_(*t*) indicate residual noise. Prior to model estimation, time series data were resampled to 5 Hz (40-point down-sampling using the “decimate” function of MATLAB to have appropriate time intervals of the estimation (the MATLB’s “decimate” function applies a 30th order, low-pass finite impulse response (FIR) filter to the original time series for data resampling). The linear trend of the data was excluded using the “detrend” function in MATLAB (see above for the information of MATLAB’s “detrend” function). The AR order (*N*), which indicates the time length of the history, was selected by minimizing the AIC (Akaike’s information criterion) in the range from 1 to 20. At the next step, the AR was set to 3 to exclude its effect on the statistical evaluation and avoid overfitting; the averaged AIC change in the AR order from 3 to 4 was less than one-tenth of the averaged AIC in the AR order from 1 to 2 and therefore, we considered the AR order 3 to be sufficient for the purpose of the present analysis. Based on the MVAR model, the power spectrum of the two time series data was estimated by the sum of the contribution of the x-specific noise (i.e., |*α*(*f*)|^2^ *σ*_*ux*_^2^) and that of y-specific noise (i.e., |*β*(*f*)|^2^ *σ*_*uy*_^2^). NCR_*y*→*x*_(*f*) was calculated by the Eq in (4) where *α*(*f*) and *β*(*f*) are frequency response functions, derived from Fourier transformation via an impulse response function, using a set of AR coefficients. *σ*_*ux*_ and *σ*_*uy*_ were noise variance of *u*_*x*_(*t*) and *u*_*y*_(*t*), respectively. NCR_*x*→*y*_(*f*) was calculated by the Eq in (5) where *γ*(*f*) and *δ*(*f*) represent the frequency response functions similar to *α*(*f*) and *β*(*f*). The NCR was mathematically integrated by a trapezoidal numerical integration and produced one ΣNCR value (see the Eq in (6)). (See [33] for the details of the analysis method and equations adopted here.)

$$x(t)=\sum_{i=1}^{N}a_{i}x(t-i)+\sum_{i=1}^{N}b_{i}y(t-i)+u_{x}(t)$$ (2)

$$y(t)=\sum_{i=1}^{N}c_{i}x(t-i)+\sum_{i=1}^{N}d_{i}y(t-i)+u_{y}(t)$$ (3)

$${\mathrm{NCR}}_{y\to x}(f)=\frac{{\left|\beta (f)\right|}^{2}{\sigma_{uy}}^{2}}{{\left|\alpha (f)\right|}^{2}{\sigma_{ux}}^{2}+{\left|\beta (f)\right|}^{2}{\sigma_{uy}}^{2}}$$ (4)

$${\mathrm{NCR}}_{x\to y}(f)=\frac{{\left|\gamma (f)\right|}^{2}{\sigma_{ux}}^{2}}{{\left|\delta (f)\right|}^{2}{\sigma_{uy}}^{2}+{\left|\gamma (f)\right|}^{2}{\sigma_{ux}}^{2}}$$ (5)

$$\sum {NCR}_{y\to x}=\int_{0}^{fs/2}{NCR}_{y\to x}(f)df$$ (6)

The ΣNCR was analyzed with three-way repeated measures ANOVAs with factors SENDER (Open, Blindfold), RECEIVER (Open, Blindfold), and DISTANCE (Near, Far). The first two factors, SENDER and RECEIVER, represent the direction (the start and end point) of visual information flow, with both SENDER and RECEIVER being either eyes open or blindfolded. This statistical design was adopted since, as mentioned, the causality analysis investigates the degree of influence on postural sway from one person to another, and importantly, we were interested in testing whether or not the degree of influence changes depending on the direction of visual information sent or received between the paired participants (i.e., SENDER, RECEIVER). The statistical analyses were conducted for AP and LP axes, separately.

#### Single regression analysis of the results in cross correlation and causality analysis

We investigated whether the degree of the causal influence between two participants (e.g., one participant contributing more than the other) is related to the occurrence of the time lag of the synchronization for postural sway. To investigate this, we focused on the OO condition, and the relationship between the ΣNCR difference of paired participants and the time lag in the synchronization was assessed across all pairs of participants using a single regression analysis (Eq (7)).

$$Y_{n}=B_{0}+B_{1}X_{n}$$ (7)

In the Eq in (7), X_*n*_ and Y_*n*_ is the difference in ΣNCR and time lag of the synchronization for each pair of participants, respectively. B_0_ and B_1_ indicate the intercept and slope of the regression, respectively.

#### Model simulation

**Fig 2:**
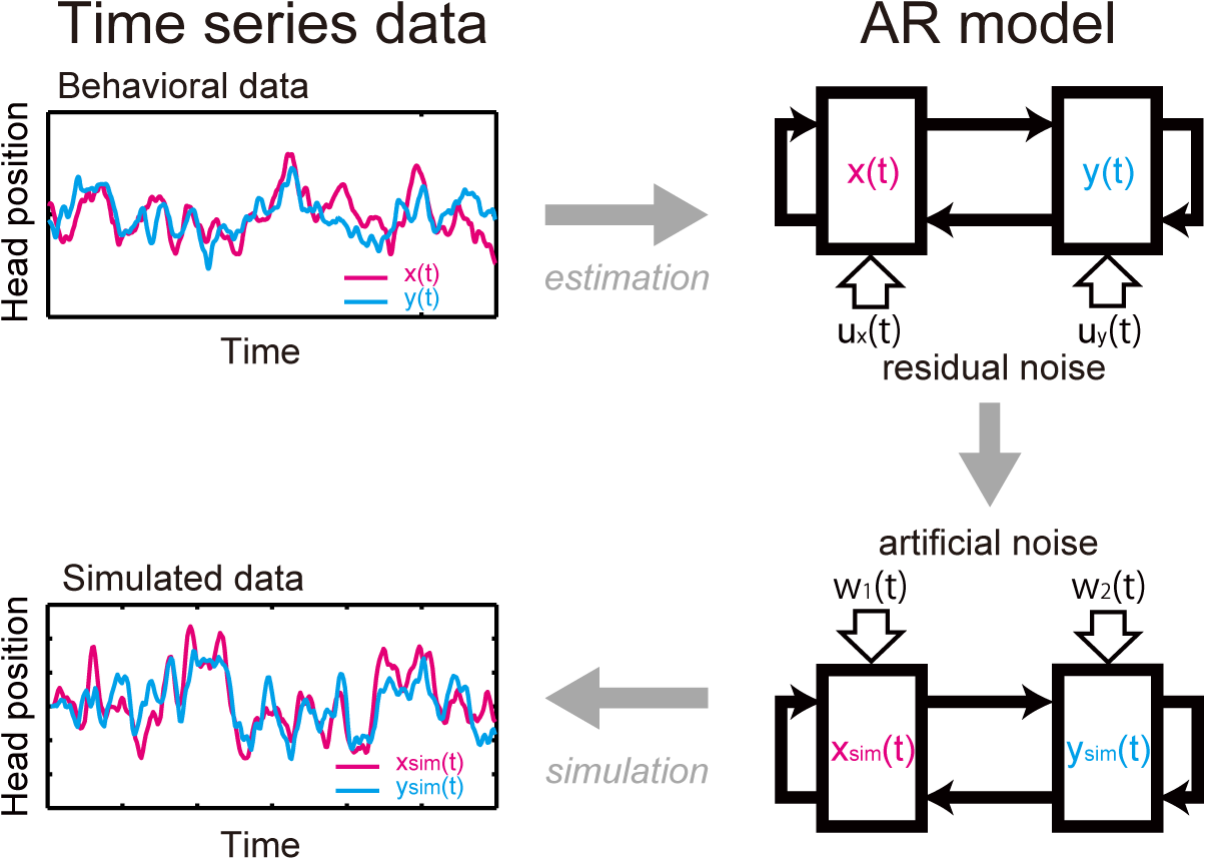
Simulation procedures. The upper figures show a model estimation process for a bi-variate AR model (upper right figure) based on the time series data of paired participants’ postural sway (i.e., their head position) obtained in the behavioral experiment (upper left figure). “x” and “y” represent each of the paired participants in the behavioral experiment, and “x(*t*)” and “y(*t*)” correspond to the time series data of their postural sway. The lower figures show a model simulation process to generate simulated postural sway data (lower left figure) using the bi-variate AR model (lower right figure). “x_*sim*_(*t*)” and “y_*sim*_(*t*)” represent simulated time series data for the postural sway of paired participants. During the simulation process, the residual noise (*u*_*x*_(*t*) and *u*_*y*_(*t*)) incurred as a result of the model estimation of the AR-model (upper right figure) was replaced with artificial noise (*w*_1_(*t*) and *w*_2_(*t*)) (lower right figure). The artificial noise was used as driving input to generate the simulated data of postural sway of paired participants. The simulation process was repeated 40 times with 40 different sets of artificial noise over the model parameters based on four trials, each with four VISUAL INTERACTION conditions (see Fig 1), carried by 22 paired participants in the behavioral experiment.

Virtual postural sway between virtually created paired participants (x_*sim*_(*t*) and y_*sim*_(*t*)) was simulated using empirically pre-estimated AR coefficients obtained from the OO, BO, and OB conditions in the reported behavioral study (see above) and artificial noise input (Fig 2). This was done to further investigate the relationship between interpersonal influence and the time lag of postural sway synchronization. Importantly, such a simulation allowed us to systematically evaluate the validity of our proposed model with a wider range of input. The artificial noise input was made from a white noise *w*_*i*_(*t*) (i.e., *w*_1_(*t*) and *w*_2_(*t*) in Eqs (8) and (9)) produced by the MATLAB’s function, “randn” (the MATLAB’s “randn” function generates random numbers in a normally distribution with the mean = 0 and standard deviation = 1). The virtual postural sway (x_*sim*_(*t*) and y_*sim*_(*t*)) was computed using the different pairs of artificial noise input for each of the three conditions of VISUAL INTERACTION (OO, BO, and OB) (Eqs (8) and (9)). The simulation was repeated 40 times for each of the virtually created paired participants, for the same number of trials and conditions as the actually conducted behavioral experiment. An analysis of cross correlation was performed for the simulated data, just like the behavioral experiment (see the Eq in (1) above). The mean cross correlation was calculated and the time lag of the virtual postural sway synchronization was detected. For each of the three conditions of VISUAL INTERACTION (OO, BO, and OB), we checked the relationship between the time lag of behavioral data and that of simulated data using a single regression analysis (Eq (7), with X and Y, the time lag of simulated and those of behavioral data, respectively). Then, the results of time lag of simulated data in the OO condition was tested against 0, using a one-sample *t*-test. The time lag results of simulated data in the BO and OB conditions were tested against those in the OO condition using paired *t*-tests. In both single regression analysis and *t*-tests, *p*-values were Bonferroni corrected (*p*-values in the results indicate corrected *p*-values unless otherwise noted).

$$x_{sim}(t)=\sum_{i=1}^{N}a_{i}x(t-i)+\sum_{i=1}^{N}b_{i}y(t-i)+w_{1}(t)$$ (8)

$$y_{sim}(t)=\sum_{i=1}^{N}c_{i}x(t-i)+\sum_{i=1}^{N}d_{i}y(t-i)+w_{2}(t)$$ (9)

## Results

### Questionnaire Results Supporting the Performed Behavioral Task

Of the 24 participants that filled out the optional questionnaire, almost all (23 out of 24) were aware of their own postural sway, and 75% (18 out of 24) were also aware of their partner’s postural sway. However, none of these participants noticed that their postural sway was synchronized with that of their partner. All participants that filled out the questionnaire reported that they did not attempt to either synchronize or asynchronize their postural sway with their partner. These results suggest that the experiment was conducted as we intended and support the findings that the synchronization of postural sway between pairs of participants occurred unintentionally.

### Time Lag of the Postural Synchronization Revealed by Cross Correlation

**Fig 3:**
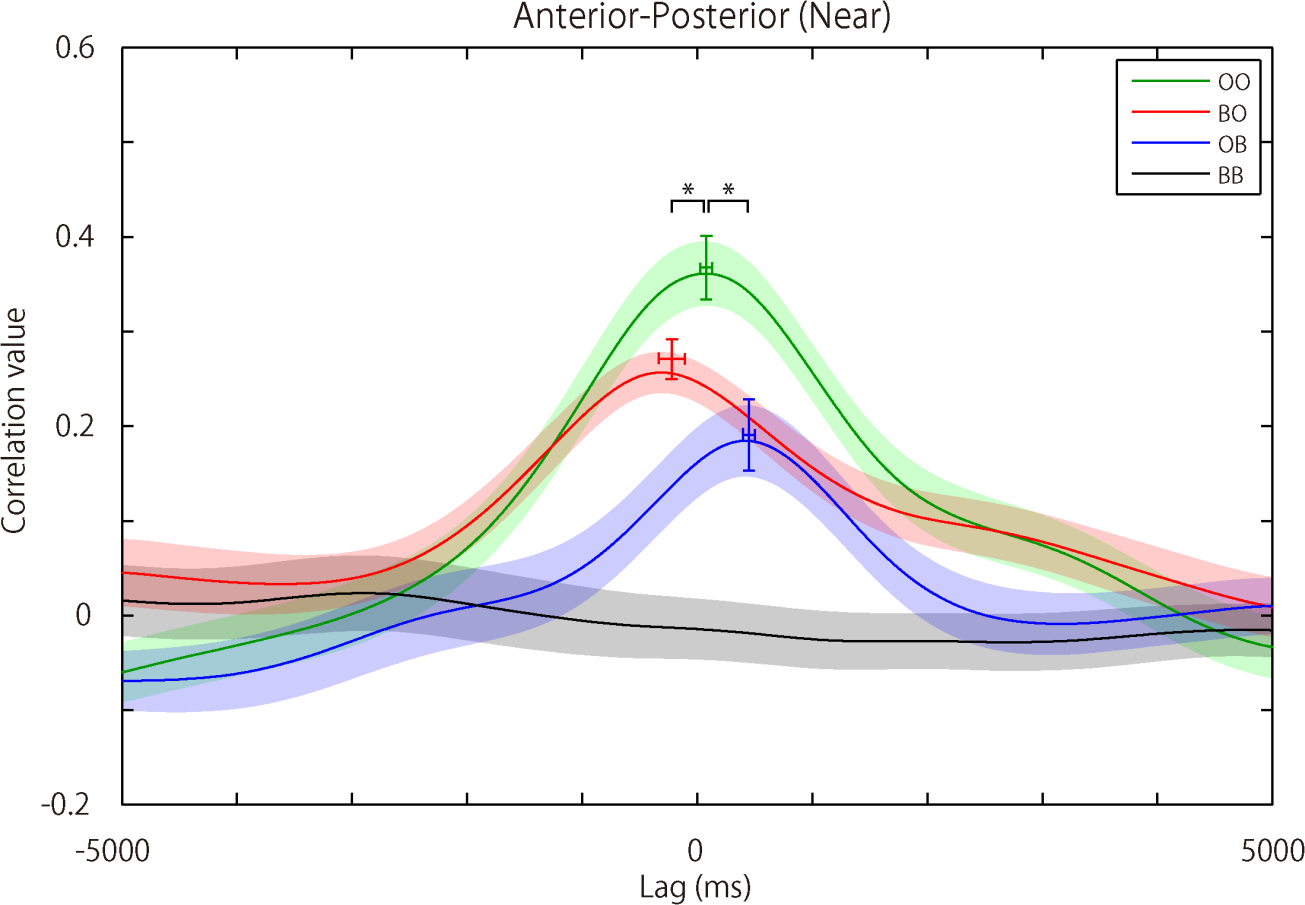
Cross correlation results for participants’ postural sway. The figure shows grand averaged cross correlation curves for participants’ postural sway data for each of the VISUAL INTERACTION conditions (OO, BO, OB, and BB) (see Fig 1 for the test conditions). Curves are plotted for postural sway along an Anterior-Posterior (AP) axis and for the Near DISTANCE condition only. The x-axis represents “time lag (ms)” centered around 0 up to ± 5000 ms, and the y-axis represents the “correlation value”. The solid lines indicate the means of each condition and the pale color areas standard error of the mean (SEM). Colored crosses over the lines indicate the peak of the curves at the time point around 0 ms, before and after 0 ms, with respect to the lag-0 time point (0 ms) for the OO, BO, and OB conditions, respectively. The horizontal line of the cross indicates SEM for the time lag of the peaks. The vertical line of the cross indicates SEM for the amplitude (correlation value) of the peaks. This suggests full synchronization of postural sway (with no time lag) between paired participants when they both had their eyes open (the OO condition). When only one of the paired participants had her eyes open, postural sway was synchronized, but occurred slightly earlier (the BO condition) or later (the OB condition) than the lag-0 time. The BB condition had no apparent peak in the correlation curve, showing that postural sway did not synchronize when paired participants were both blindfolded. (See S1 Fig) for the results for the Near DISTANCE condition along a Left-Right (LR) axis as well as the results for the Far DISTANCE condition along both AP and LR axes).

Postural coordination of an AP axis for the Near DISTANCE condition showed a time lag in the OO condition of 78 ± 239 ms (mean ± SD). This result was not significantly different from 0 ms (*p* = 0.140). In contrast, for the same axis (AP) and DISTANCE (Near) conditions, both BO and OB conditions showed a significantly and systematically different time lag (Open participants delayed) from that in the OO condition (BO: -220 ± 529 ms, *p* = 0.016; OB: 448 ± 254 ms, *p* < 0.001) (see Fig 3). These results imply that lag-0 synchronization (i.e., no time lag in postural sway synchronization between two participants) occurred when two participants both had their eyes open. They also suggest that when one of the participants was blindfolded (BO or OB condition), postural sway of the blindfolded participants preceded that of their sighted partners. Postural sway along the LR axis for the Near DISTANCE condition showed results similar to those of the AP axis: Time lag in the OO condition was not significant against 0 ms (OO: 51 ± 201 ms, *p* = 0.244) and the time lag in the BO and OB conditions was significantly different against that in the OO condition (BO: -305 ± 491 ms, *p* = 0.013; OB: 309 ± 390 ms, *p* = 0.033). The Far DISTANCE condition for both AP and LR axes showed the patterns of results similar to those of the Near DISTANCE conditions (see S1 Fig); however, nothing approached significance, and the condition-specific time lag differences were not clear (For AP, OO: 19 ± 446 ms, *p* = 0.843; BO: 115 ± 784 ms, *p* = 0.617 (uncorrected); OB: -62 ± 692 ms, *p* = 0.645 (uncorrected); for LR, OO: -15 ± 428 ms, *p* = 0.873; BO: -130 ± 534 ms, *p* = 0.425 (uncorrected); OB: 57 ± 718 ms, *p* = 0.720 (uncorrected)). (See S1 Table for the signal variance of postural sway in all conditions.) For the full datasets of postural sway, see S1 Data (for the Near Distance condition) and S2 Data (for the Far Distance condition).

### Interpersonal Influence on Postural Sway Synchronization

**Fig 4:**
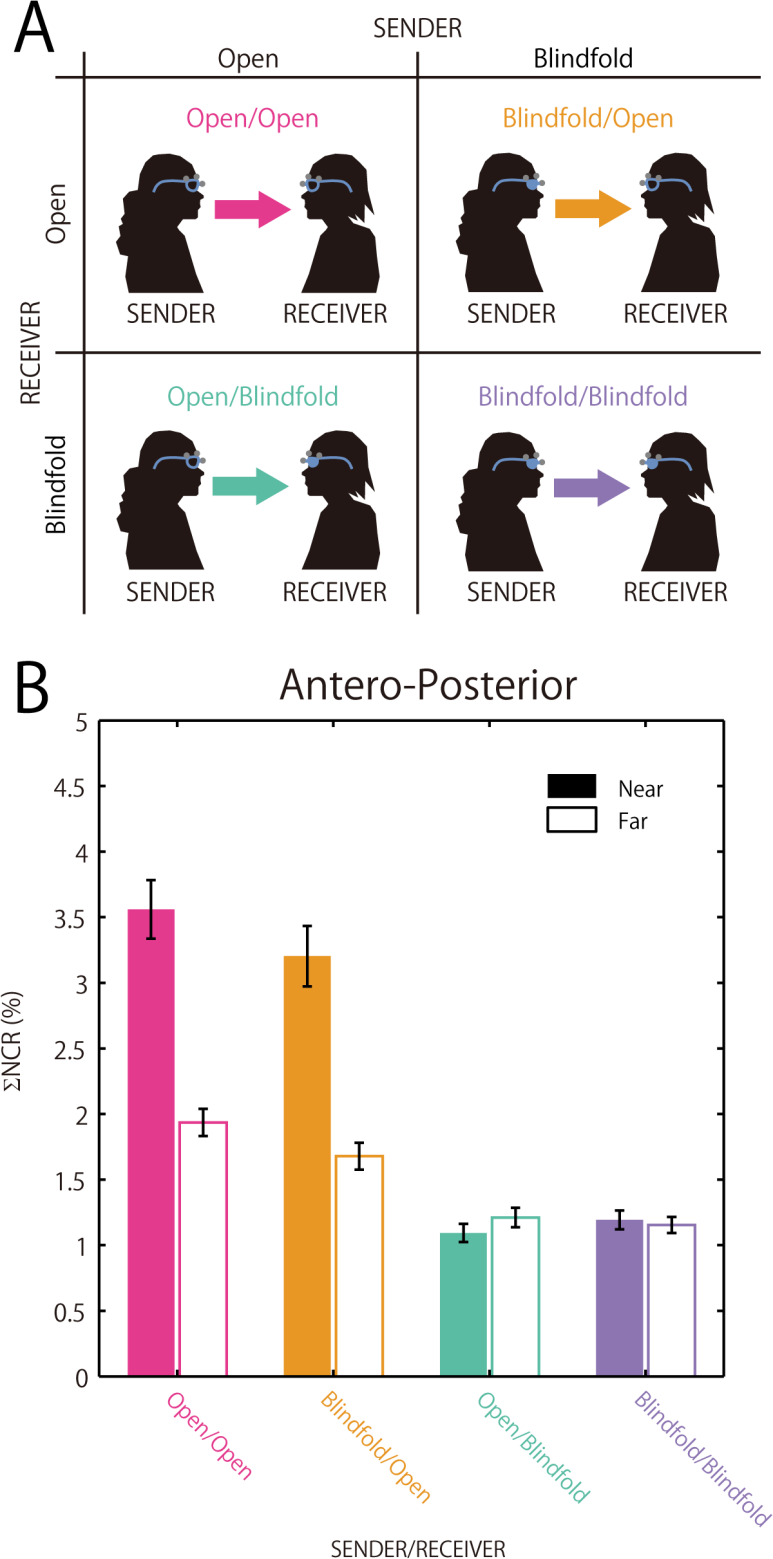
Magnitude of influence on participants’ postural sway and its relation to visual state. (A) The figure illustrates the design of the analysis conducted. (Note: Only two factors (SENDER vs. RECEIVER, each with Open or Blindfold, are shown. The third factor, DISTANCE is not). SENDER and RECEIVER correspond to the direction of the exchange of visual information (participants serving the role of sending visual information and the role of receiving it, respectively). For each role, the participants were either sighted (Open) or blindfolded (Blindfold). (B) ΣNCR (%) along the y-axis shows the magnitude of influence on postural sway between paired participants. The direction of the exchange of visual information (SENDER vs. RECEIVER) for the four experimental conditions is indicated on the x-axis (see (A)). The left side of each of the paired visual states separated by slashes corresponds to SENDER and the right side RECEIVER (e.g., in case of Blindfold/Open, the SENDER is blindfolded and the RECEIVER is sighed). The dark bars show the results for the Near DISTANCE conditions and the light bars the results for the Far DISTANCE conditions. The results are plotted for the postural sway along the anterior-posterior axis only. The figure illustrates a significant interaction between the direction of information flow (SENDER vs. RECEIVER) and DISTANCE (Near vs. Far). The amount of influence the RECEIVER received was significantly larger when the SENDER had eyes Open than Blindfolded (compare the left two cases, i.e., Open/Open and Blindfold/Open against the right two cases, i.e., Open/Blindfold and Blindfold/Blindfold). In addition, participants showed the aforementioned postural sway influence to a larger degree in the Near DISTANCE conditions than in the Far DISTANCE conditions (compare the dark bars in the left two states against the corresponding light bars). See Table 1 for the statistical results.

**Fig 5:**
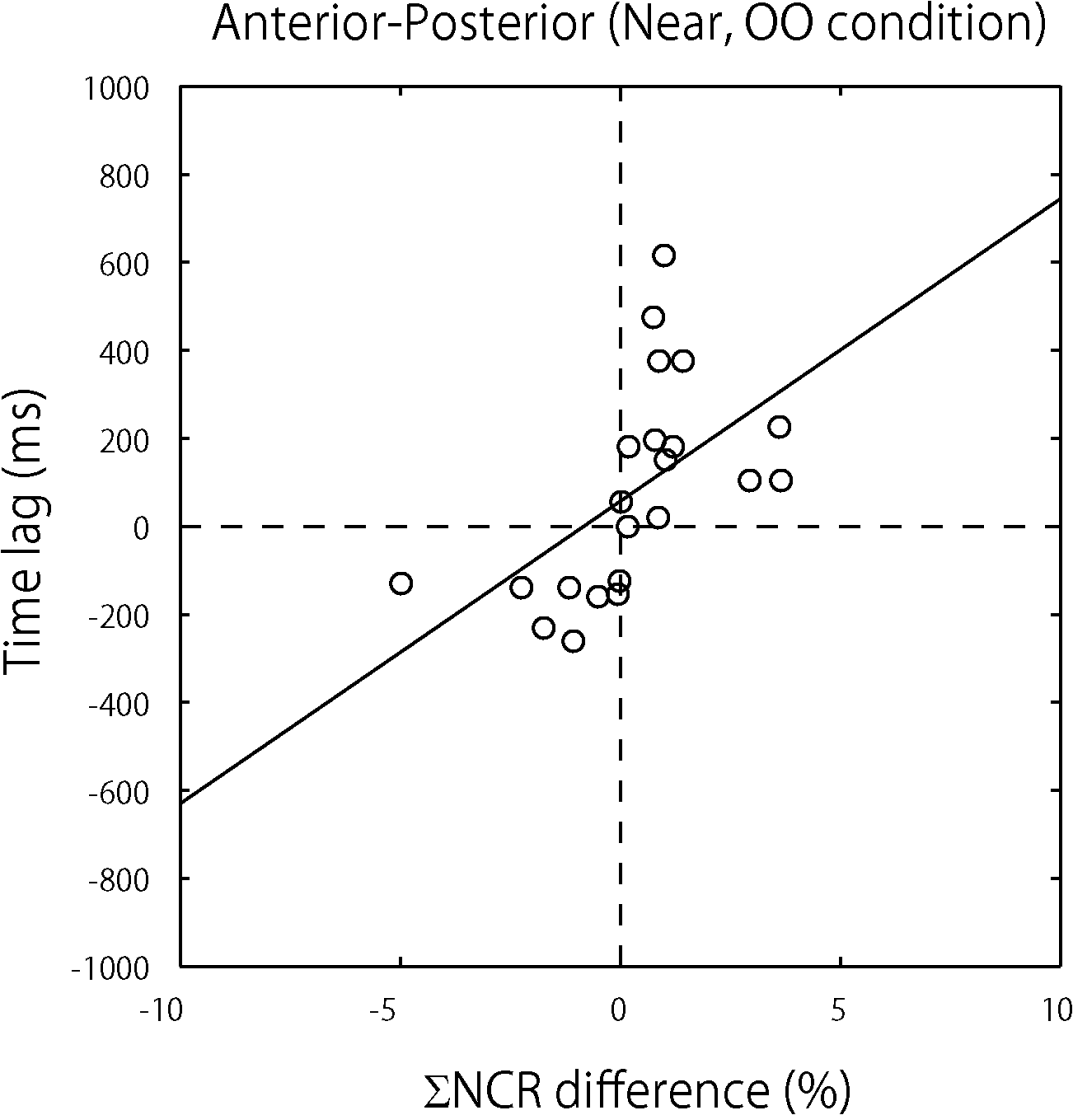
Relation between participants’ influence and time lag for postural sway synchronization. Each dot in the figure represents the average of all trials for each of the paired participants. The data are plotted in the following way. X-axis values represent differences in paired participants’ influence on one another’s postural sway (estimated by ΣNCR difference (%)), and y-axis values represent the time lag (ms) that occurred when postural sway was synchronized. These data come from the Open-Open condition (see Fig 1 for the test conditions) and account for postural sway along an anterior-posterior axis only. As the figure shows, when the time lag is closer to 0, the ΣNCR difference (%) is closer to 0, i.e., the level of influence that paired participants exerted on one another was close to the same. This observation was supported by the regression analysis between the two factors (see the regression line included in the figure).

A three-way repeated measures ANOVA with factors SENDER (Open, Blindfold), RECEIVER (Open, Blindfold) and DISTANCE (Near, Far) was carried out for LR and AP axes, separately (for the statistical results, see Table 1; for the results for an AP axis, see Fig 4). For both AP and LP axes, there was a significant interaction between SENDER and RECEIVER and between RECEIVER and DISTANCE (see Table 1(A)). Because of the significant interactions, two-way repeated measures ANOVAs with the factors DISTANCE and SENDER were conducted for Open and Blindfolded RECEIVER for both AP and LP axes. For the AP axis, effects of SENDER and DISTANCE were significant for Open RECEIVER (see Table 1(B)). The significant effect of SENDER found here implies that paired participants being OPEN, i.e., both receiver and sender being sighted, exert great influence on each other in postural sway synchronization. As for the significant effect of DISTANCE, such a result suggests greater importance for the receiver role (i.e., the receiver’s contribution to postural synchronization) in the Near DISTANCE condition than in the Far DISTANCE condition, for open, as opposed to blindfolded, sender. This pattern of the results was also supported by the results of a simple regression analysis between the difference in ΣNCR for paired participants and the time lag in their postural synchronization (see Fig 5). (Note: For the LR axis, the Blindfolded RECEIVER showed a significant interaction with SENDER and DISTANCE. This effect seems to be a random effect, considering all other results reported in this paper. However, it should be noted that further investigation is needed.)

**Table 1:** Statistical results for the integrated noise contribution ratio (ΣNCR).

**A**	**Three-way ANOVA (AP)**				**Three-way ANOVA (LR)**			
**Effect**	***df***	***F*-value**	***p*-value**	***η***_***P***_^***2***^	***df***	***F*-value**	***p*-value**	***η***_***P***_^***2***^
SENDER	1,43	2.95	. 093	. 064	1,43	7.93	. 007**	. 156
RECEIVER	1,43	133.14	<.001***	. 756	1,43	101.89	<.001***	. 703
DISTANCE	1,43	87.18	<.001***	. 670	1,43	51.39	<.001***	. 544
SENDER×RECEIVER	1,43	4.29	. 044*	. 091	1,43	9.68	. 003**	. 184
SENDER×DISTANCE	1,43	<1	. 856	. 001	1,43	<1	. 774	. 002
RECEIVER×DISTANCE	1,43	73.51	<.001***	. 631	1,43	53.25	<.001***	. 553
SENDER×RECEIVER×DISTANCE	1,43	<1	. 420	. 015	1,43	3.73	. 060	. 080
**B**	**Following two-way ANOVA (AP)**				**Following two-way ANOVA (LR)**			
**RECEIVER: Open**								
**Effect**	***df***	***F*-value**	***p*-value**	***η***_***P***_^***2***^	***df***	***F*-value**	***p*-value**	***η***_***P***_^***2***^
SENDER	1,43	4.34	. 043*	. 092	1,43	10.88	. 002**	. 202
DISTANCE	1,43	93.39	<.001***	. 685	1,43	60.28	<.001***	. 584
SENDER×DISTANCE	1,43	<1	. 730	. 003	1,43	<1	. 403	. 016
**RECEIVER: Blindfold**								
**Effect**	***df***	***F*-value**	***p*-value**	***η***_***P***_^***2***^	***df***	***F*-value**	***p*-value**	***η***_***P***_^***2***^
SENDER	1,43	<1	. 755	. 002	1,43	<1	. 974	<.001
DISTANCE	1,43	<1	. 567	. 008	1,43	<1	. 892	<.001
SENDER×DISTANCE	1,43	1.52	. 225	. 034	1,43	6.13	. 017*	. 125

(A) Global ANOVAs for postural sway along anterior-posterior and left-right axes. (B) Post-hoc ANOVAs for the RECEIVER condition for Open and Blindfold.

***, ** and * in the tables indicate *p*-values < 0.001, < 0.01, and < 0.05, respectively. Partial eta-squared (*η*_*P*_^*2*^) represents the effect size.

### Relation between Participants’ Bi-Directional Influence and Time Lag for Postural Sway Synchronization

A simple regression analysis between the difference in ΣNCR for paired participants and the time lag in postural sway synchronization was carried out. The analysis was conducted for the OO condition (i.e., when both participants were sighted) in the Near DISTANCE condition (see Fig 5 for the results). The difference in ΣNCR between paired participants showed a significant positive slope with the time lag in postural sway synchronization (B_1_ = 68.71, *t* = 2.956, *p* = 0.008) but not for its intercept (B_0_ = 56.86, *t* = 1.291, *p* = 0.212). This result demonstrates that equivalent influence on participants induced lag-0 synchronization, and that the more the participants were influenced by their partners, the longer the time lag.

### Simulation Based on a Multivariate Autoregressive Model

**Fig 6:**
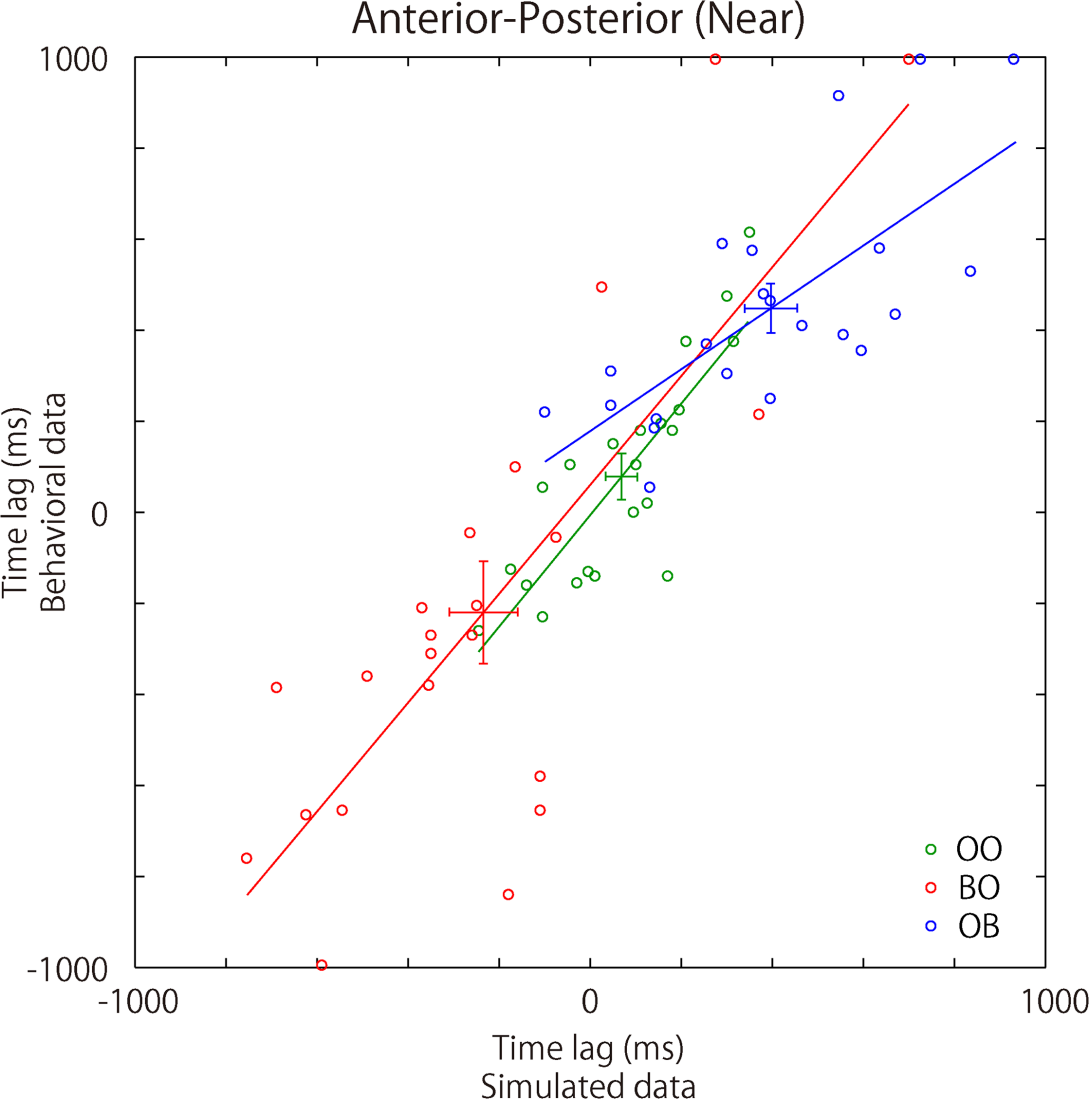
Simulated results for time lag in synchronization. The figure illustrates the relationship between the synchronization time lag for the simulated and the behavioral data (for the simulation procedures, see Fig 2). The colored dots, green, red, and blue, in the figure correspond to the results for the Open-Open, Blindfold-Open, and Open-Blindfold conditions, respectively. The same color was used for the regression line for each of the conditions. The slope for each regression line was significantly higher than 0, suggesting a positive relation between the simulated and behavioral data.

The experimental results of the time lag in postural sway along the AP axis for the Near DISTANCE condition were replicated in our simulation. The time lag of postural sway synchronization in the simulated data was correlated with that in the behavioral data across pairs of participants in the OO, BO, and OB conditions (OO: B_1_ = 1.23, *t* = 6.866, *p* < 0.001; B_0_ = -6.37, *t* = 0.206, *p* = 0.839 (uncorrected); BO: B_1_ = 1.20, *t* = 5.912, *p* < 0.001; B_0_ = 61.07, *t* = 0.723, *p* = 0.478; OB: B_1_ = 0.68, *t* = 4.700, *p* < 0.001; B_0_ = 178.26, *t* = 2.586, *p* = 0.018). In addition, the time lag in the OO condition was 68 ± 163 ms (mean ± SD). This result was not significantly different from 0 ms (*p* = 0.061). The time lag in both BO and OB conditions was significantly different from that in the OO condition (BO: -235 ± 353 ms, *p* < 0.001; OB: 397 ± 271 ms, *p* < 0.001) (see Fig 6). These simulated results are consistent with the results of the behavioral experiment reported above and reinforce our proposal about lag-0 synchronization of postural sway between two individuals.

## Discussion

### Lag-0 Synchronization

We found lag-0 synchronization in the postural sway of sighted individuals who were standing face to face. In previous studies, synchronization has been observed in the postural and manual movements of individuals participating in cooperative tasks [27,38] and rhythmic movements (e.g., [5,14,15,26]). More recently, Yun et al. (2012) [39] reported lag-0 synchronization of non-rhythmic movements when paired participants pointed and held their index fingers towards one another. As the pointing was done consciously, the participants in this study can be viewed as having shared a common goal. In our study, lag-0 synchronization of postural sway occurred unconsciously (without intent) and without any shared goal or cooperative task. (As noted earlier, no explicit instruction concerning the performed task was given to the participants; they were not instructed to move or not to move their body during the experiment.) In addition, the present study demonstrated that the reciprocity of visual information is critical for lag-0 synchronization, i.e., lag-0 synchronization occurs between sighted pairs of individuals. In other cases, either a time lag in the postural sway synchronization or no synchronization of postural sway occurs. Similar to our study, Noy et al. (2011) [27] showed the importance of the reciprocity of visual information for lag-0 synchronization. In their study, joint improvisation by expert actors and musicians created complex motions that were synchronized to less than 40 ms. To account for the observed phenomenon, they proposed a mirrored reactive-predictive controller system, in which two controllers, each of which tracks the leader’s input and output and the follower’s movement. Their system was configured like a mirror such that the output velocity of one controller served as the input for the other. While the single controller system showed some jitters (overshooting and undershooting of the output compared to the input), the mirrored configuration resulted in synchronized joint motion with no jitters. The gist of their proposal is that synchronized joint motion is a result of an implicit agreement between individuals that can predict their partner’s future motion. Different from our proposal, their proposal requires the presence of a reactive-predictive controller and its learning process. As discussed below, the reciprocal feedback system proposed in this study is simple yet captures synchronization that is unpredictable and is a result of the task performed by individuals unintentionally.

### Mechanism for Lag-0 Synchronization and Its Evidence

We proposed a reciprocal visuo-motor feedback system that accounts for lag-0 synchronization. The essence of our proposed system lies in the crucial relation between time lag in postural synchronization and the amount of visual information which individuals receive from their partners. To test our proposed system, both a behavioral experiment and a simulation with a wider range of input were carried out. The results of the reported behavioral experiment showed that the difference in the NCR calculated for each pair of the participants was positively correlated with the time lag in the postural sway synchronization (see Fig 5 above). In support of our proposal, the balanced relation in the amount of visual influence on paired participants led to lag-0 synchronization. The causality evaluated by the NCR represented the dynamics of the real-time mutual interaction between the persons standing face to face. It has been stressed that “with any phenomenological time series model, we should not jump into any conclusion without confirming the whiteness and mutual independence of the driving noise” [33]. Therefore, we conducted a simulation by reconstructing the time series data and utilizing the estimated AR parameters with newly generated paired, white and driving noises, which were independent to each other. This simulation replicated the findings of the reported behavioral study (see Fig 6 above).

### Non-Linear Interaction

The postural sway, observed as a result of the reported experiment, showed that the degree of influence calculated by means of the NCR differed between participants who were both sighted (i.e., SENDER and RECEIVER being both Open) and mixed blindfolded/sighted pairs (i.e., SENDER and RECEIVER being either Blindfold/Open or Open/Blindfold) (see Fig 4 and Table 1). Specifically, the feedback system involved in the postural sway synchronization in this study was more efficient, triggering lag-0 synchronization, when both participants were sighted, compared to the cases in which one of the participants was blindfolded. This finding indicates that the feedback system operating in postural sway is non-linear in a sense that the degree of influence on sighted participants (i.e., Open/Open) cannot be explained by the summation of the degree of influence on the sighted participants in the conditions in which only one of the participants was sighted (i.e., Blindfold/Open and Open/Blindfold). The non-linearity described here may be interpreted as a result of the individual’s unconscious sensitivity to another individual’s postural sway. Such sensitivity to one’s partner’s postural sway might have contributed to the increased efficiency of the feedback system in the two sighted partner case. The non-linearity observed in the present study results may be related to contingency detection between one’s own behavior and a consequential social event, which is often absent in disorders such as autism spectrum disorder [40,41]. Another possibility might be that in postural sway, which is subjected to higher variability [30,31], the blindfolded person might have less impact on her sighted partner. These and other possible accounts should be investigated.

### Functional Implication of “Causality”

We suggest the maintenance of personal space likely plays a role in one’s postural sway. Specifically, the maintenance of one’s personal space seems to have a significant impact on the synchronization of postural sway along the AP axis. In the reported behavioral experiment, when one of the paired participants swayed forward, her partner swayed back, and vice versa. This suggests that maintaining an appropriate degree of personal space [42] may be one of the driving forces of the causality. This interpretation is supported by the fact that causality was larger in the Near DISTANCE condition than in the Far DISTANCE condition. In the Near DISTANCE condition, the personal space of participants was more closely co-located, and so possibly more “repulsive force”.

It is well documented that movement of visual surroundings induces body displacement in the same direction as that of the visual stimuli [29,43–45]. Visual surroundings may also serve as one of the driving forces for the causality. The postural sway along the LP axis, though not restricted to the LP axis, may be a good example here. In the left-right axis, postural sway occurred in the same direction between paired participants. In other words, the postural sway of the paired participants occurred in a mirror image, i.e., one of the paired participants moved to her right and then her partner to her left, and vice versa. Since visual proprioceptive information is generally more sensitive if the person is facing a nearby object [44], in the face to face standing condition, visual input signaling the partner’s body or head position perhaps plays a direction-specific role in the unconscious (or unintentional) control of postural sway. This applies to the synchronization of postural sway along both AP and LP axes. Interesting future research includes questioning whether and if so how factors such as personal space and surrounding visual input modulate the synchronization of postural sway in more dynamic social interactions between individuals.

## Conclusions

This paper demonstrated that reciprocal exchange of visual information results in lag-0 synchronization of the postural sway between sighted participants standing face to face. The experimental paradigm reported in this paper provides an empirical method for exploring the physiological basis for lag-0 synchronization. In addition, the present study showed that an MVAR model, specifically Akaike causality, serves, as a useful tool for quantifying the degree of contribution of individuals taking part in synchronized postural sway. We believe that the findings in the reported experiment and the proposed mechanism for lag-0 synchronization can be extended to other synchronized movements and events that are observed in daily life.

## Supporting Information

S1 Data Full datasets for the postural sway in the behavioral experiment (Near Distance condition). The file includes the postural sway data (in MATLAB format) for all participants for the Near Distance condition in the reported behavioral study.
(ZIP)

S2 Data Full datasets for the postural sway in the behavioral experiment (Far Distance condition). The file includes the postural sway data (in MATLAB format) for all participants for the Far Distance condition in the reported behavioral study.
(ZIP)

S1 Fig Cross correlation results for participants’ near distance postural sway along a left-right axis and both near and far distances along both anterior-posterior and left-right axes. Three figures above show grand averaged cross correlation curves for participants’ postural sway for each of the VISUAL INTERACTION conditions (OO, BO, OB, and BB) (see Fig 1 for the test conditions) for the Near DISTANCE condition along a Left-Right (LR) axis (see (A)) and the results for the Far DISTANCE condition along both AP and LR axes (see (B) and (C)). See Fig 3 for information about how the figures were plotted.
(TIF)

S1 Table Signal variance of postural sway. The units used in the table are mm. AP, LR, and RC stand for Anterior-Posterior, Left-Right, and Rostro-Caudal axes, respectively. Near and Far correspond to Near and Far DISTANCE tested in the reported experiment. The notation used for the conditions works as follows: "O (with partner: B)" means the signal variance (or the amplitude) in the postural sway for the (Eyes-)Open participants when their partners were blindfolded.
(TIF)

## References

[1] Haken H. Advanced synergetics: Instability hierarchies of self-organizing systems and devices Berlin: Springer-Verlag; 1983.

[2] Nicolis G, Prigogine I. Self-organization in non-equilibrium systems New York: Wiley; 1977.

[3] Bernieri F, Rosenthal R. Interpersonal coordination: Behavior matching and interactional synchrony In: Feldman RS, Rime B, editors. Fundamentals of nonverbal behavior. New York: Cambridge University Press; 1991.

[4] Strogatz SH. Sync: The Emerging Science of Spontaneous Order. New Yotk: Hyperion; 2003.

[5] Oullier O, de Guzman GC, Jantzen KJ, Lagarde J, Kelso JA. Social coordination dynamics: measuring human bonding. Soc Neurosci. 2008;3(2):178–92. 10.1080/17470910701563392 18552971 PMC2156197

[6] Chartrand TL, Bargh JA. The chameleon effect: the perception-behavior link and social interaction. J Pers Soc Psychol. 1999;76(6):893–910. 10402679 10.1037//0022-3514.76.6.893

[7] Dimberg U, Lundquist LO. Gender differences in facial reactions to facial expressions. Biol psychol. 1990;30(2):151–9. 2285765 10.1016/0301-0511(90)90024-q

[8] LaFrance M. Nonverbal Synchrony and Rapport: Analysis by the Cross-Lag Panel Technique. Soc Psychol Q. 1979;42(1):66–70.

[9] Lakin JL, Jefferis VE, Cheng CM, Chartrand TL. The chameleon effect as social glue: Evidence for the evolutionary significance of nonconscious mimicry. J Nonverbal Behav. 2003;27(3):145–62.

[10] Meltzoff AN, Moore MK. Imitation of facial and manual gestures by human neonates. Science. 1977;198(4312):75–78. 17741897 10.1126/science.198.4312.75

[11] Hattori Y, Tomonaga M, Matsuzawa T. Spontaneous synchronized tapping to an auditory rhythm in a chimpanzee. Sci Rep. 2013;3:1566 10.1038/srep01566 23535698 PMC3610097

[12] Todt D, Marc N. Vocal interactions in birds: the use of song as a model in communication. Adv Study Behav 2000;29:247–95.

[13] Bailenson JN, Beall AC, Loomis J, Blascovich J, Turk M. Transformed social interaction: Decoupling representation from behavior and form in collaborative virtual environments. PRESENCE: Teleoperators and Virtual Environments. 2004;13(4):428–41.

[14] Richardson MJ, Marsh KL, Isenhower RW, Goodman JRL, Schmidt RC. Rocking together: dynamics of intentional and unintentional interpersonal coordination. Hum Mov Sci. 2007;26(6):867–91. 17765345 10.1016/j.humov.2007.07.002

[15] Schmidt RC, O’Brien B. Evaluating the dynamics of unintended interpersonal coordination. Ecol Psychol. 1997;9(3):189–206.

[16] Konvalinka I, Vuust P, Roepstorff A, Frith CD. Follow you, follow me: continuous mutual prediction and adaptation in joint tapping. Q J Exp Psychol. 2010;63(11):2220–30. 10.1080/17470218.2010.497843 20694920

[17] Miles LK, Nind LK, Macrae CN. The rhythm of rapport: Interpersonal synchrony and social perception. J Exp Soc Psychol. 2009;45(3):585–9.

[18] Hove MJ, Risen JL. It’s All in the Timing: Interpersonal Synchrony Increases Affiliation. Soc Cogn. 2009;27(6):949–60.

[19] Macrae CN, Duffy OK, Miles LK, Lawrence J. A case of hand waving: Action synchrony and person perception. Cognition. 2008;109(1):152–6. 10.1016/j.cognition.2008.07.007 18755450

[20] Shockley KD, Santana MV, Fowler CA. Mutual interpersonal postural constraints are involved in cooperative conversation. J Exp Psychol Hum Percept Perform. 2003;29(2):326–32. 12760618 10.1037/0096-1523.29.2.326

[21] Shockley KD, Richardson DC, Dale R. Conversation and Coordinative Structures. Top Cogn Sci. 2009;1(2):305–19. 10.1111/j.1756-8765.2009.01021.x 25164935

[22] De Jaegher H, Di Paolo E, Gallagher S. Can social interaction constitute social cognition? Trends Cogn Sci. 2010;14(10):441–7. 10.1016/j.tics.2010.06.009 20674467

[23] Frith CD. Making Up the Mind: How the Brain Creates Our Mental World. New York, New York, USA: Blackwell Science; 2007.

[24] Hari R, Kujala MV. Brain basis of human social interaction: from concepts to brain imaging. Physiol Rev. 2009;89(2):453–79. 10.1152/physrev.00041.2007 19342612

[25] Marsh KL, Richardson MJ, Baron RM. Contrasting approaches to perceiving and acting with others. Ecol Psychol. 2006;18(1):1–38.

[26] Sebanz N, Bekkering H, Knoblich G. Joint action: bodies and minds moving together. Trends Cogn Sci. 2006;10(2):70–6. 16406326 10.1016/j.tics.2005.12.009

[27] Noy L, Dekel E, Alon U. The mirror game as a paradigm for studying the dynamics of two people improvising motion together. Proc Natl Acad Sci U S A. 2011;108(52):20947–52. 10.1073/pnas.1108155108 22160696 PMC3248496

[28] Bronstein AM. Suppression of visually evoked postural responses. Exp brain Res. 1986;63(3):655–8. 3489640 10.1007/BF00237488

[29] Bronstein a. M, Buckwell D. Automatic control of postural sway by visual motion parallax. Exp Brain Res. 1997;113:243–8. 9063710 10.1007/BF02450322

[30] Diener HC, Dichgans J, Bootz F, Bacher M, Gompf B. Early stabilization of human posture after a sudden disturbance: influence of rate and amplitude of displacement. Exp Brain Res. 1984;56(1):126–34. 6468561 10.1007/BF00237448

[31] Paulus WM, Straube A, Brandt T. Visual stabilization of posture. Physiological stimulus characteristics and clinical aspects. Brain. 1984;107(4):1143–63. 6509312 10.1093/brain/107.4.1143

[32] Akaike H. On the use of a linear model for the identification of feedback systems. Ann Inst Stat Math. 1968;20:425–39.

[33] Ozaki T. Time series Modeling of Neuroscience Data. FL: CRC press; 2012.

[34] Wong KFK, Ozaki T. Akaike causality in state space. Instantaneous causality between visual cortex in fMRI time series. Biol Cybern. 2007;97(2):151–7. 17579884 10.1007/s00422-007-0165-1

[35] Dijkstra TMH, Schiiner G, Giese MA, Gielen CCAM. Frequency dependence of the action-perception cycle for postural control in a moving visual environment: relative phase dynamics. Biol Cybern. 1994;501:489–501. 10.1007/BF00198467 7999875

[36] Oie KS, Kiemel T, Jeka JJ. Multisensory fusion: Simultaneous re-weighting of vision and touch for the control of human posture. Cogn Brain Res. 2002;14(1):164–76. 10.1016/s0926-6410(02)00071-x 12063140

[37] Baccalá LA, Sameshima K. Partial directed coherence: a new concept in neural structure determination. Biol Cybern. 2001;84(6):463–74. 11417058 10.1007/PL00007990

[38] Ramenzoni VC, Davis TJ, Riley MA, Shockley KD, Baker A. Joint action in a cooperative precision task: nested processes of intrapersonal and interpersonal coordination. Exp Brain Res. 2011;211(3–4):447–57. 10.1007/s00221-011-2653-8 21479660

[39] Yun K, Watanabe K, Shimojo S. Interpersonal body and neural synchronization as a marker of implicit social interaction. Sci Rep. 2012;2(959):1–8. 10.1038/srep00959 PMC3518815 23233878

[40] Gergely G. The Obscure Object of Desire: “Nearly, but Clearly Not, Like Me”: Contingency Preference in Normal Children Versus Children with Autism. Bull Menninger Clin. 2001;65:411–26. 11531136 10.1521/bumc.65.3.411.19853

[41] Okamoto Y, Kitada R, Tanabe HC, Hayashi MJ, Kochiyama T, Munesue T, et al Attenuation of the contingency detection effect in the extrastriate body area in autism spectrum disorder. Neurosci Res. 2014;87:66–76. 10.1016/j.neures.2014.06.012 25066523

[42] Kennedy DP, Gläscher J, Tyszka JM, Adolphs R. Personal space regulation by the human amygdala. Nat Neurosci. 2009;12(10):1226–7. 10.1038/nn.2381 19718035 PMC2753689

[43] Dichgans J, Brandt T. Visual-vestibular interaction: effects on self-motion perception and postural control. Perception (Handbook of sensory physiology, vol VIII). 1978. p. 755–804.

[44] Lee DN, Lishman JR. Visual proprioceptive control of stance. J Hum Mov Stud. 1975;1:87–95.

[45] Lestienne F, Soechting J, Berthoz A. Postural readjustments induced by linear motion of visual scenes. Exp brain Res. 1977;28(3–4):363–84. 885185 10.1007/BF00235717

