# Supplementary figures and images for: Unintentional Interpersonal Synchronization Represented as a Reciprocal Visuo-Postural Feedback System: A Multivariate Autoregressive Modeling Approach
Source: PLoS One. 2015 Sep 23;10(9):e0137126. doi: 10.1371/journal.pone.0137126 (PMC4580648; doi:10.1371/journal.pone.0137126)

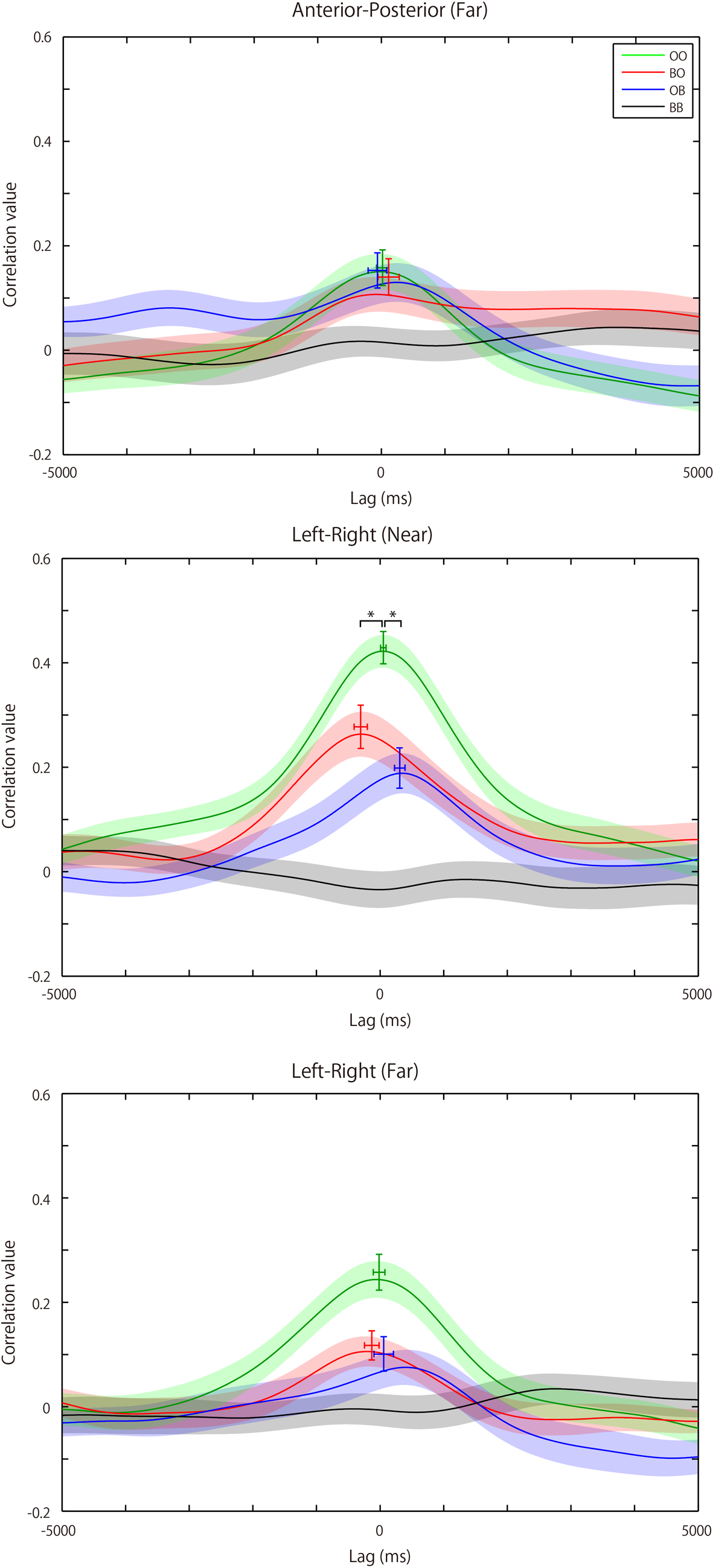

Supplement: S1 Fig — Three figures above show grand averaged cross correlation curves for participants’ postural sway for each of the VISUAL INTERACTION conditions (OO, BO, OB, and BB) (see Fig 1 for the test conditions) for the Near DISTANCE condition along a Left-Right (LR) axis (see (A)) and the results for the Far DISTANCE condition along both AP and LR axes (see (B) and (C)). See Fig 3 for information about how the figures were plotted. (TIF) [file pone.0137126.s003.tif]

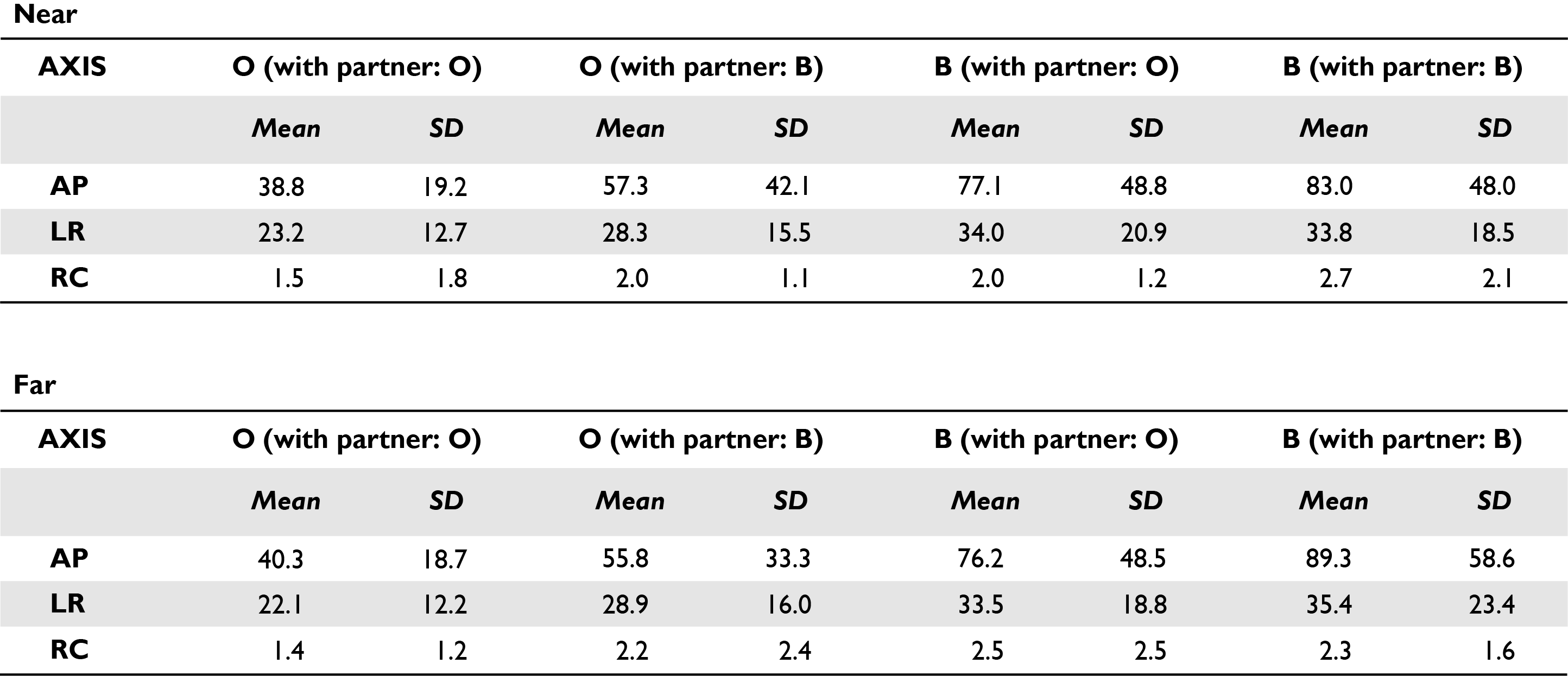

Supplement: S1 Table — The units used in the table are mm. AP, LR, and RC stand for Anterior-Posterior, Left-Right, and Rostro-Caudal axes, respectively. Near and Far correspond to Near and Far DISTANCE tested in the reported experiment. The notation used for the conditions works as follows: "O (with partner: B)" means the signal variance (or the amplitude) in the postural sway for the (Eyes-)Open participants when their partners were blindfolded. (TIF) [file pone.0137126.s004.tif]
